# Prediction of global left ventricular functional recovery in patients with heart failure undergoing surgical revascularisation, based on late gadolinium enhancement Cardiovascular Magnetic Resonance

**DOI:** 10.1186/1532-429X-12-56

**Published:** 2010-10-07

**Authors:** Tammy J Pegg, Joseph B Selvanayagam, Joslin Jennifer, Jane M Francis, Theodoros D Karamitsos, Erica Dall'Armellina, Karen L Smith, David P Taggart, Stefan Neubauer

**Affiliations:** 1 University of Oxford Centre for Clinical Magnetic Resonance Research, (OCMR), UK; 2 Nuffield Department of Surgery, University of Oxford, UK; 3 Flinders Medical Centre, Adelaide, South Australia; 4 Centre for Statistics in Medicine, University of Oxford, UK

## Abstract

**Background:**

The new gold standard for myocardial viability assessment is late gadolinium enhancement-cardiovascular magnetic resonance (LGE-CMR); this technique has demonstrated that the transmural extent of scar predicts segmental functional recovery. We now asked how the number of viable and number of viable+normal, segments predicted recovery of global left ventricular (LV) function in patients undergoing CABG. Finally, we examined which segmental transmural threshold of scarring best predicted global LV recovery.

**Methods and Results:**

Fifty patients with reduced LV ejection fraction (EF) referred for CABG were recruited, and 33 included in this analysis. Patients underwent CMR to assess LV function and viability pre-operatively at 6 days and 6 months. Mean LVEF 38% ± 11, which improved to 43% ± 12 after surgery. 21/33 patients improved EF by ≥3% (EF before 38% ± 13, after 47% ± 13), 12/33 did not (EF before 39% ± 6, after 37% ± 8). The only independent predictor for global functional recovery after revascularisation was the number of viable+normal segments: Based on a segmental transmural viability cutoff of <50%, ROC analysis demonstrated ≥10 viable+normal segments predicted ≥3% improvement in LVEF with a sensitivity of 95% and specificity of 75% (AUC = 0.9, p < 0.001). Transmural viability cutoffs of <25 and <75% and a cutoff of ≥4 viable segments were less useful predictors of global LV recovery.

**Conclusions:**

Based on a 50% transmural viability cutoff, patients with ≥10 viable+normal segments improve global LV function post revascularisation, while patients with fewer such segments do not. LGE-CMR is a simple and powerful tool for identifying which patients with impaired LV function will benefit from CABG.

**Trial registration:**

Research Ethics Committee Unique Identifier: NRES:05/Q1603/42. The study is listed on the Current Controlled Trials Registry: ISRCTN41388968.

URL: http://www.controlled-trials.com

## Background

In some patients with coronary artery disease and impaired left ventricular (LV) function, revascularisation by coronary artery bypass grafting (CABG) improves both symptoms and prognosis[1], while in the absence of significant viability, revascularisation offers little prognostic benefit[2]. Several studies have directly linked post-surgical improvement in LV ejection fraction (EF) and symptoms to the presence of significant viable myocardium[3-6]. Viability testing is now an integral part of the assessment of patients with impaired LV function and coronary artery disease considered for revascularisation[7].

Over the last decade, LGE-CMR has emerged as a simple and highly reproducible tool for assessing both myocardial injury and viability. The seminal work by Kim *et al.*[5] demonstrated that the transmural extent of myocardial injury predicted regional functional recovery on a segmental level. However, even more important than the issue of segmental recovery is the question of identifying which patients with poor LV function will show recovery of global LV function after revascularisation, and which will not. Studies using positron emission tomography (PET) and Dobutamine Stress Echo (DSE) have started to define the numbers of viable segments, based on a 16 segment model, associated with global functional recovery. Bax *et al. *demonstrated functional recovery in patients with 4 or more viable segments on DSE, with an approximate sensitivity of 84% and specificity of 81%[8]. More recently Slart *et al.*[9] showed that 3 or more viable segments defined by FDG uptake by PET, predicted global functional recovery with a sensitivity and specificity of 87% and 85%, respectively. However, unlike LGE-CMR, these methods cannot define the segmental transmural extent of scar. With LGE-CMR increasingly becoming the gold standard for viability imaging, it is important to understand how both the number of viable segments and the transmural extent of viability, assessed by LGE-CMR, predicts global recovery of LV function.

We have reported the results of a randomised trial comparing cardioplegic arrest CABG (ONSTOP) to a novel method for intra-operative myocardial protection (on-pump beating heart, ONBEAT) [10]. This study used CMR to image patients with heart failure undergoing CABG before, at 6 days and at 6 months after surgery. Using data from this unique cohort, we asked two simple, practical questions: Based on a 16 segment AHA model (omitting the true apex), does the sum of viable segments or the sum of viable+normal segments provide the stronger cutoff criteria for global LV recovery? Secondly, what cutoff for segmental transmural extent of viability best predicts recovery?

## Methods

The methods have been described in detail before[10]. Patients with impaired LV function accepted for surgery were recruited if they consented and had no contra-indications to CMR or gadolinium contrast. Recruited patients included both elective admissions and patients with recent unstable coronary syndromes requiring inpatient revascularisation; patients with Class IVb angina were excluded. All elective patients were assessed with CMR within 4 weeks of their surgery, whilst all urgent in-hospital referrals for CABG underwent their pre-operative CMR assessment the evening before surgery. This study complies with the Declaration of Helsinki, a locally appointed ethics committee had approved the research protocol (NRES:05/Q1603/42), all patients gave written informed consent.

### Treatment and Procedures

The aim of CABG was to obtain complete revascularisation, all territories were assumed to be revascularised, intra operative graft imaging was routinely undertaken.

### CMR protocol

All CMR examinations were performed using a 1.5 Tesla MR scanner (Sonata, Siemens Medical Solutions, Erlangen, Germany), using prospective gating. After piloting, steady-state free precession cine images (temporal resolution 24 - 45 ms; TE/TR 1.5/3.0 ms, flip angle 60^o^) were acquired. The short axis stack was acquired parallel to the AV groove in 1 cm increments (slice thickness 7 mm, inter-slice gap 3mm).

LGE-CMR was performed with a T1-weighted segmented inversion-recovery turbo fast low-angle shot (FLASH) sequence (echo time 4.8ms, voxel size 1.4 × 2.4 × 7 mm, flip angle 20°) following a 6 minute time delay after the administration of 0.1 mmol/kg contrast agent (Gadodiamide, Omniscan™, GE Healthcare). The inversion time was meticulously adjusted for optimal nulling of remote normal myocardium.

### Post Processing Analysis

The methods for analysing and calculating LV volumes and LGE are standardised within our unit and, along with the reproducibility, have been published [11,12]. Left ventricular volumetric analysis used the short axis ventricular stack and was analysed using Argus software (Version 2002B, Siemens Medical Solutions) by a single experienced observer (J.M.F) blinded to RWM and LGE findings. Manual tracing of endocardial borders in each successive slice position at the chosen end diastolic and end systolic phase was performed. The basal slice was selected if its circumference comprised at least 50% of myocardium. Papillary muscle was included in the LV mass and excluded from the LV volume. Visual assessment of regional wall motion score (RWMS) using Argus software (Version 2002B, Siemens Medical Solutions) was undertaken by two experienced observers working in consensus and blinded to the LGE findings. Segments were graded 1-normally contracting to 5-dyskinetic. Improvement in regional contraction was defined by an improvement of ≥1 functional grade (with the exception of improvement from grade 5 to grade 4).

Transmural extent of myocardial infarction was quantified by a computer assisted planimetry programme, MATLAB version 7.3.0.267 (Natick USA) by a single experienced operator, with areas of myocardial infarction determined as areas with signal intensity >2 S.D above remote normal myocardium. Semi-quantitative approach with operator override was employed to categorize the transmural extent of hyperenhancement into subgroups: 0 = no LGE, 1 = 1-25% LGE, 2 = 26-50% LGE, 3 = 51-75% LGE and 4 > 75% LGE. Inter-observer variability for transmurality grading was checked for 15 patients by a second blinded observer, with a kappa = 0.872, indicating a good level of agreement. To calculate the mass of LGE, we assumed a specific gravity of 1.05 g/cm^3^.

### Registration of segments

For visual assessment of transmural extent of scar and RWM, two models, based on either a 48 segment (6 slices × 8 segments) or the AHA 16 segment model (excluding segment 17 - apex) was used. The basal slice was defined as the first slice without LVOT in any phase of the cardiac cycle. Segment 1 was defined at the anterior insertion of the right ventricle into the inter-ventricular septum. Registration of segments for LGE-CMR and regional wall motion scores was undertaken in a paired manner by a single observer 6 months prior to visual analysis. For the 16 segment AHA model, the mid-ventricular slice was defined as 20 mm below the base, on condition that it contained papillary muscle but no trabeculation, similarly the apical slice was defined as 20 mm below the mid ventricular slice on condition that it contained trabeculation but no papillary muscle.

### Statistical Analysis

There was an excess in myocardial injury associated with the ONBEAT technique, therefore we excluded patients with evidence of new myocardial injury based on their early post operative scan or on the presence of significantly elevated troponin levels following surgery (in patients not able to complete imaging at 6 days). The main comparisons of the study are between patients with functional recovery and patients without improvement in LVEF.

Values were expressed as mean (SD) or median (inter-quartile range, 25% to 75%). The effect of revascularisation was compared using a paired t-test and dichotomous data was compared by the χ^2 ^statistic or Fisher's exact test. Continuous variables that were not distributed normally were compared by the Mann-Whitney test.

To compare the transmural extent of scar and regional functional recovery, a logistic generalised estimating equation model accounting for non-independence of the data within each subject was used. To examine the impact of the number of non-viable segments on regional recovery in viable segments, a similar logistic generalised estimating equation model was used, adjusting for baseline LGE, surgical technique, ESVI and EF. For both analyses, both the independent (no adjustments made for within subject correlation of the data) and AR1 models (accounting for within subject correlation in the data) were examined with no effect on the results. The AR1 model is reported. A probability of p < 0.05 was considered statistically significant.

Due to the number of independent variables involved in determining late LV recovery, we adopted a model building strategy to assess the potential association between baseline variables and change in EF at 6 months. We first performed a simple regression analysis to examine any potential association between the baseline variables (i.e. age, sex, BMI, ONBEAT versus ONSTOP, number of grafts, total mass of LGE, % Left anterior descending artery (LAD) territory affected by >50% LGE, number of viable segments, number of viable+normal segments, time between surgery and CMR scan, pre-operative LVEF and ESVI) and late change in EF. Variables with p < 0.1 were then included in the multiple linear regression using an enter selection method to assess the best factors for predicting change in LV function.

Receiver Operating Characteristic (ROC) analysis was performed to determine the number of viable segments, and the number of viable+normal segments, that best predicts recovery of global function, where improvement is defined as ≥3%[13] absolute change in LVEF.

## Results

**Figure 1:**
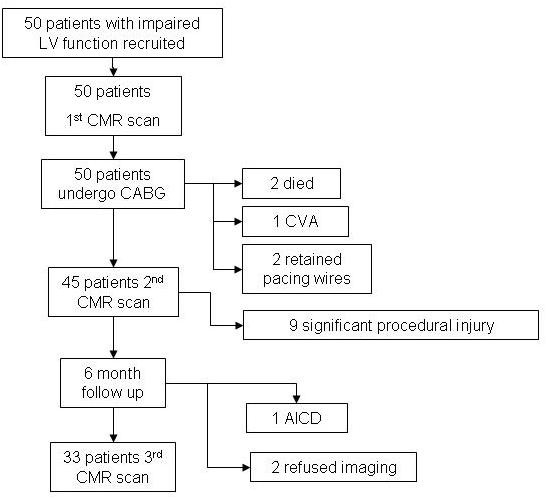
**Consort statement diagram of trial participants**. AICD, automated implantable cardiac defibrillator; CABG, coronary artery bypass grafting; CMR, cardiovascular magnetic resonance imaging; CVE, cerebro-vascular accident; LV, left ventricular.

Patients recruited and excluded from this study are presented in Figure 1. Data analysis is based on 33 patients. Mean pre-operative LVEF for patients was 38% ± 11, mean pre-operative regional wall motion score (RWMS) was 2.4 ± 0.7. Eight patients had recent evidence (< 28 days) of acute coronary syndrome. There was an excellent linear relationship between baseline EF and mean RWMS for each individual patient (r = 0.85, p < 0.0001).

All patients recruited had three vessel coronary artery disease or left main stem disease. The median number of grafts per patient was 4(3-4), all grafts were patent at the end of surgery. All patients were reviewed at 6 months, no patient had recurrent angina or an intervening hospital admission.

### Prediction of regional segmental functional recovery

**Figure 2:**
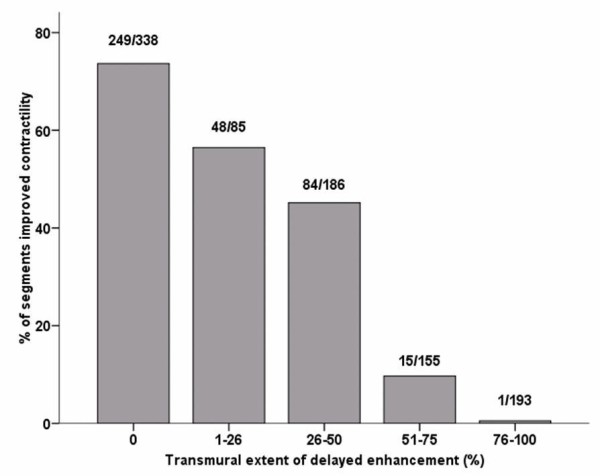
**Relationship between the transmural extent of scar and functional recovery on a segmental basis**.

Prior to defining predictors of global recovery we wanted to ensure that the previously described relationship between transmural extent of viability and regional function recovery holds true for this cohort of patients with more severely impaired LV function. For this purpose it was necessary to use a 40 or 48 segment model similar to previous work[5,14]. A total of 1408 segments were available for analysis, of which 957 segments were judged to be dysfunctional before revascularisation (68%). Furthermore, 718 segments had some degree of LGE (51%). In dysfunctional segments, there was progressive reduction in functional recovery with increasing extent of transmural infarction (p < 0.001) (Figure 2).

**Figure 3:**
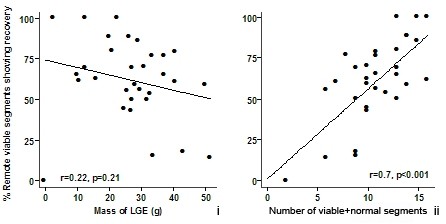
**Panel i Correlation between recovery of remote and adjacent viable segments and the mass of late gadolinium enhancement (LGE) before surgery**. Panel ii Correlation between recovery of remote and adjacent segments with the number of viable+normal segments.

Overall, patients improved their LVEF from 38% ± 11 to 43% ± 12 (p < 0.001). Of dysfunctional segments affected by <50% LGE, 381/609 (63%) improved contraction by 6 months. Segments with <50%LGE were considered "viable" for this purpose. In patients, the number of viable and viable+normal (<50% scar) segments, not the mass of LGE, had linear relationships with the percentage of viable segments that demonstrated recovery of function (Figure 3i and ii). This association was maintained after adjusting for baseline ESVI, EF, surgical technique and the extent of LGE in these viable segments (p < 0.001).

### Patient response to coronary artery bypass grafting

An improvement in EF of ≥3% was demonstrated in 21/33 patients (64%), and the characteristics of patients with and without significant improvement in LVEF are given in Table 1.

**Table 1:** Patient demographics

	All patients (n = 33)	Responders (n = 21)	Non-responders (n = 12)	p value
**Age**	66 ± 8	67 ± 8	63 ± 8	0.15
**GFR**	65 ± 16	66 ± 18	64 ± 11	0.83
**BSA(m^2^)**	2 ± 0.2	2 ± 0.2	2 ± 0.1	0.83
**ONBEAT**	12	5	7	
**ONSTOP**	21	16	5	0.07
**B Blocker**	25	15	10	0.574
**ACE inhibitor**	27	12	19	0.493
**AUC troponin**	215	265	156	0.89
**(0-120 h)**	(109-347)	(103-346)	(109-412)	
**Numbers of distal**	4	3.5	4	0.31
**anastomoses**	(3-4)	(2.25-4)	(3-4)	
**Duration between surgery and final CMR**	217 ± 23	225 ± 29	204 ± 22	P = 0.04
**EF(%)**	38 ± 11	38 ± 13	39 ± 6	0.77
**EDVI(ml.m^-2^)**	118 ± 33	120 ± 34	116 ± 32	0.76
**ESVI(ml.m^-2^)**	75 ± 32	77 ± 36	72 ± 27	0.65
**Mass of LGE(g)**	28 ± 12	27 ± 11	28 ± 15	0.83
**Viable+normal segments**	11 ± 3	12 ± 2	8 ± 3	< 0.001
**6 month EF(%)**	43 ± 12	47 ± 13	37 ± 8	0.02

Responder is defined as improvement in EF (EF) ≥3%. BSA, body surface area (m^2^); LGE, late gadolinium enhancement; EDVI, end diastolic volume index; ESVI, end systolic volume index; ONBEAT, on-pump beating heart coronary artery bypass grafting; ONSTOP, conventional cardioplegic arrest coronary artery bypass grafting.

### Prediction of global functional recovery based on the 16 segment AHA model

**Figure 4:**
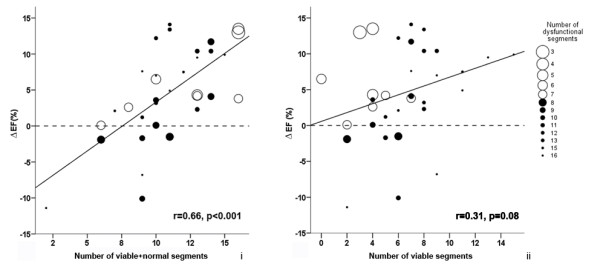
**Panel i. Correlation between the number of viable+normal segments and change in EF at 6 months (Δ EF)**. Panel ii. Scatter plot showing the relationship between the number viable segments and change in EF at 6 months.

The relationship between the number of viable+normal segments (< 50% transmural scar) (Figure 4, panel i) and change in EF was linear. A lesser relationship was demonstrated when the number of viable segments was considered (Figure 4, panel ii). Using a multivariate model, only the sum of viable+normal segments (but not the number of viable segments) was shown to independently predict change in EF 6 months following revascularisation (Table 2 and Figure 4, panel i).

**Table 2:** Univariate and multivariate analyses for the prediction of global functional recovery following coronary artery bypass grafting.

	Univariate analysis		Multivariate analysis	
	
	r	p	Beta	p
**Number of viable+normal segments**	0.67	< 0.001	1.298	< 0.001
**Number of viable segments**	0.31	0.08		
**% of LAD territory viable**	0.35	0.05		
**Mean duration to 3rd CMR (days)**	0.35	0.04		

Number of viable segments was defined as segments <50% transmural extent of late gadolinium enhancement. CMR, cardiovascular magnetic resonance; LAD, left anterior descending coronary artery.

**Figure 5:**
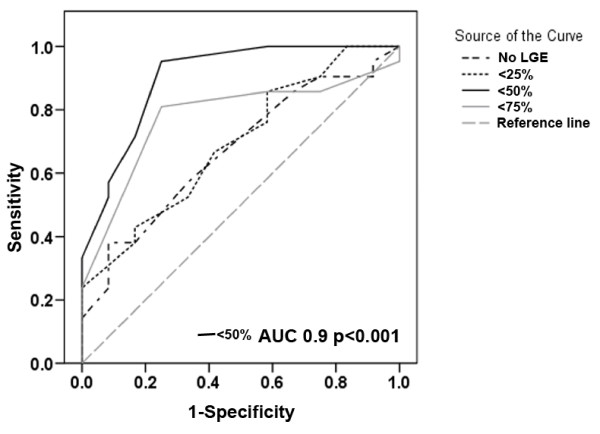
**ROC analysis for the threshold of viable segments that predict global functional recovery**. Legend shows various transmural extent of LGE. Optimal diagnostic performance was achieved with ≥10 viable+normal segments (segments affected by <50% LGE).

ROC analysis was used to best define a threshold for number of viable+normal segments and also the transmural extent of LGE within each segment that had the optimal sensitivity and specificity for predicting global functional recovery (Figure 5). For improvement in LVEF, 10 or more viable+normal segments (LGE <50%) demonstrated the optimal sensitivity of 95% and specificity of 75%. Furthermore, both the positive and negative predictive values were high (87% and 89% respectively) (AUC 0.90 p < 0.001, Figure 5). A transmural extent of LGE <50% was the best threshold to define segmental viability for the purpose of predicting global recovery, while other transmural cutoff thresholds were less powerful.

Patients with ≥10 viable+normal segments on LGE-CMR demonstrated both significant improvement in EF and reverse remodeling, whereas there was no evidence of a change in overall function or LV geometry at 6 months in patients with <10 viable+normal segments (Table 3).

**Table 3:** Remodeling in patients subsequent to surgery.

	Pre-op	6 months	Mean difference	p value
**≥10 segments viable+normal (n = 22)**				

**EDVI(ml.m^-2^)**	116 ± 32	105 ± 32	12 ± 23	0.03
**ESVI(ml.m^-2^)**	74 ± 32	59 ± 29	15 ± 17	0.001
**EF(%)**	39 ± 11	46 ± 11	7 ± 5	p < 0.001

**<10 segments viable+normal (n = 11)**				

**EDVI(ml.m^-2^)**	122 ± 35	119 ± 38	2 ± 10	0.50
**ESVI(ml.m^-2^)**	79 ± 34	79 ± 39	-1 ± 15	0.87
**EF(%)**	38 ± 11	37 ± 12	1 ± 6	0.59

EF, ejection fraction; ESVI, end systolic volume index; EDVI, end diastolic volume index. Viable defined as <50% transmural extent of late gadolinium enhancement in each segment.

**Figure 6:**
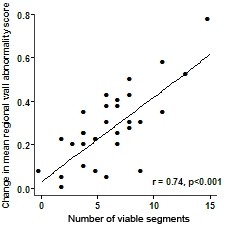
**Correlation between the mean improvement in mean regional wall motion score and the number of viable segments**.

The number of viable segments correlated very well with the mean change in regional wall motion score after CABG (Figure 6) but yielded a lower sensitivity and specificity for predicting global functional recovery. Four or more viable segments predicted ≥3% improvement in LVEF with a sensitivity of 76%, specificity of 42% (AUC 0.722, p = 0.04), clearly inferior to the number of viable+normal segments.

Finally, in line with convention we also examined a 5% threshold for improvement in LVEF, where 10 or more viable+normal segments had a sensitivity of 93%, specificity 49%, positive predictive value 56%, negative predictive value 90% and AUC 0.75, p = 0.02. Six patients who were defined at the 3% level for improvement in LVEF did not meet criteria for improvement at the 5% level. In these intermediate patients, ΔEF was +4 ± 0.6%, ΔEDVI -16 ± 15 ml.m^-2^, ΔESVI -13 ± 10 ml.m^-2^.

## Discussion

The most important findings of this single centre study are: Using a 16 segment model and a definition of segmental viability equaling <50% of transmural extent, the number of viable+normal segments demonstrated a linear relationship with improvement in ejection fraction and was able to predict patients with an overall improvement in EF of ≥3% at 6 months with high sensitivity and specificity. Significant recovery of global function and positive remodeling was present in patients with at least 10 viable+normal segments, but not in those with less than 10 viable+normal segments. These findings are of direct clinical relevance for clinicians making decisions about revascularisation in patients with impaired LV function in everyday practice.

### Prediction of regional functional recovery

The study by Kim *et al.*[5] was first to demonstrate a progressive loss of functional recovery with increasing transmural extent of LGE. Furthermore, they demonstrated that although 79% of segments without evidence of scar had improved contractility at 3 months, still a significant proportion had not. Similar work in patients with normal pre-operative EF imaged at 6 months found a slightly higher rate of functional recovery in segments without pre-existing LGE (82%)[14]. We similarly expected that imaging later and excluding patients with procedure related injury, would again optimize the proportion of viable segments with functional recovery after CABG. However, only 73% of unscarred segments in our cohort recovered. Our group differed from both that described by Kim *et al.*[5] and Selvanayagam *et al.*[14] in that our mean LVEF was lower (38 vs. 43 or 61%), and with a greater proportion of both dysfunctional and scarred segments. With a higher prevalence of scar, our finding that the number of scarred segments affected recovery of viable segments, may explain our lower rates of regional functional recovery.

### Prediction of global functional recovery

We found that the number of viable+normal segments demonstrated a linear relationship with functional recovery and was the only independent predictor of the change in LVEF at 6 months, while the number of viable segments was not. Furthermore, other baseline variables such as mass of LGE did not demonstrate association with overall functional recovery. However, recruited patients included those with evidence of recent acute coronary syndrome, and myocardial infarction defined by CMR undergoes some degree of remodeling and contraction with age. Given the varying age of the myocardial scar in our patients, the lack of association is unsurprising.

Rizello *et al. *[15] using DSE, and Kim *et al. *[5] using LGE, first showed a linear relationship between the number of viable segments and the change in EF after revascularisation, although their analysis was not based on a 16 segment model, did not examine whether there is a cut off for the number of segments that predicted improvement of global function and did not include assessment of the sum of viable+normal segments. However, patients with documented viability often fail to recover global function subsequent to revascularisation. Studies investigating the diagnostic performance of viability assessment by different imaging modalities have shown that they are generally sensitive (81-93%) but less specific (58-80%)[16]. The lower specificity may be because of incorrect labeling of non viable segments (false positive), or for other reasons not relating to the imaging modality, including procedural injury (such patients were excluded in our study to eliminate this effect) [14], incomplete revascularisation, LV remodeling[17], and tethering by adjacent scarred segments[18].

The impact of LV remodeling on outcome after surgical revascularisation was first described by Yamaguchi *et al.*[19] They identified that significant pre-operative LV dilation (ESVI >100ml.m^-2^) was associated with a poor post-operative outcome, although no measure of viability was made in this study. Further to this, Bax *et al.*[17] suggested that in patients with substantial viability (≥25% LV), sub groups with extensive pre-operative remodeling (ESV ≥140 ml) do not show substantial improvement in LVEF following CABG. In a cohort comparable to the latter study, we found no relationship between pre-operative ESVI and change in either global or regional function after revascularisation. However, our numbers are relatively small and it is conceivable that in a larger study ESVI may emerge as a secondary determinant of change in LVEF.

Finally, our finding that the number of viable+normal segments was related to recovery in other viable segments supports the notion that tethering by scar tissue may prevent regional and global recovery.

### Analysis based on number of viable segments vs. number of viable+normal segments

Previous studies have based prediction of global recovery on the number of viable segments (defined as segments that are dysfunctional but viable, with the ability to recover after revascularisation)[3,9], but our study introduces the concept that the sum of viable+normal segments may be a better predictor. To illustrate this, let us consider two hypothetical patients. Patient A has 4 viable, 7 non-viable and 3 normal segments, while patient B has 4 viable, 0 non-viable and 10 normal segments. The number of viable segments is the same (4) for both patients, but the sum of viable+normal segments is 7 vs. 14. Intuitively, prognosis of these patients will be different post CABG. Thus, in our cohort, we analysed the predictive power of both the number of viable segments and the sum of viable+normal segments. This point is best exemplified in the current work in patients with mild LV dysfunction, few dysfunctional segments but in whom there was still a measurable improvement in LVEF at 6 months (Large circles: Figure 5, panels i and ii). Similar work from our group in patients with normal LV function showed similar improvement despite only 20% of analyzed segments being dysfunctional prior to surgery; with LVEF improving from 61% ± 11 to 67% ± 10 at 6 months. We hypothesize that visual assessment misses subtle LV systolic dysfunction, but the improvement is still detected by highly reproducible means such as volume assessment by CMR.

When examining change in regional wall motion scores, the number of viable (but not including normal) segments showed the better association. The latter is likely because analysis of change in regional wall motion score effectively excludes segments with normal baseline function, correlating only improved dysfunctional segments with the number of viable segments, hence the better association.

To include only viable segments in a reporting algorithm for overall functional improvement presumes that segments with normal baseline function sustain no further improvement after revascularisation and make no contribution to an increase in EF. Our data suggest that analysis should incorporate the sum of viable+normal, i.e. all segments able to contribute to the end-point.

Finally, although convention defined a threshold of 5% for clinically significant improvement in LVEF, this is not founded on prognostic data but more what was felt to be clinically relevant, which in the main part is determined by the reproducibility of transthoracic echo and LV angiography. CMR is able to detect a 3% change in LVEF with certainty[13]. In the current study, we found that using a 5% threshold for improvement in LVEF missed several patients with significant (>10%) reduction in overall LV cavity size. Neither the 3% nor the 5% threshold has been shown to be of prognostic importance. Furthermore, Senior *et al. *[20] determined that an improvement in LV geometry, not 5% improvement in LVEF, was associated with improved outcome after CABG, and they conclude that any degree of LV remodeling is likely to be associated with a survival benefit. Any reproducibly detectable improvement in function after CABG is likely to be relevant to patients.

## Limitations

The major limitation of this study is the small sample size, and these findings, although interesting, should be considered preliminary. Follow up angiography was not undertaken as part of this study, and long-term graft patency cannot be excluded as a confounding factor. By chance, patients with significant improvement in LVEF had a statistically greater duration between surgery and the 3^rd ^CMR scan. However, in practice, the difference was small and is unlikely to be of any clinical or biological relevance[21]. Previous studies have used an arbitrary threshold of 5%, we however chose a threshold for functional recovery based upon the reproducibility of the technique[13]. However, neither the 3% nor the 5% improvement in ejection fraction have been shown to translate to an improved outcome after surgery.

## Conclusions

Our study suggests that the presence of 10 or more viable plus normal segments, based upon the AHA 16 segment format and defined as <50% transmural scar, predicts significant long term recovery of global LV function in patients with impaired EF undergoing CABG. This finding is important, and may provide a simple approach to identify those patients who derive functional and prognostic benefit from CABG.

## Abbreviations

CABG: Coronary Artery Bypass Grafting; CMR: Cardiovascular Magnetic Resonance Imaging; DSE: Dobutamine Stress Echocardiography; EF: Ejection Fraction; ESVI: End systolic volume index; LGE: Late Gadolinium Enhancement; LV: Left ventricle; PET: Positron Emission Tomography; ROC: Receiver Operating Curves

## Competing interests

The authors declare that they have no competing interests.

## Authors' contributions

TJP carried out recruitment, analysis and drafted the manuscript. JBS assisted with the study design and was general supervisor to TJP. JJ performed data analysis. JMF acquired CMR images and performed analysis. TDK acquired CMR images and performed analysis. EDA performed data analysis. KLS performed the statistical analysis. DPT was principle investigator on the study and was general supervisor to TJP. SN was general supervisor for this work and interpreted the study findings. All authors have read and approved the contents of this manuscript.
